# SIRT1 Antagonizes Oxidative Stress in Diabetic Vascular Complication

**DOI:** 10.3389/fendo.2020.568861

**Published:** 2020-11-16

**Authors:** Teng Meng, Weifeng Qin, Baohua Liu

**Affiliations:** ^1^ Shenzhen Key Laboratory for Systemic Aging and Intervention, National Engineering Research Center for Biotechnology (Shenzhen), Medical Research Center, Shenzhen University Health Science Center, Shenzhen, China; ^2^ Key Laboratory of Optoelectronic Devices and Systems of Ministry of Education and Guangdong Province, College of Optoelectronic Engineering, Shenzhen University, Shenzhen, China; ^3^ Guangdong Key Laboratory of Genome Stability and Human Disease Prevention, Department of Biochemistry & Molecular Biology, School of Basic Medical Sciences, Shenzhen University, Shenzhen, China

**Keywords:** SIRT1, oxidative stress, diabetic vascular complications, adenosine-monophosphate-activated protein kinase, mechanistic target of rapamycin, microRNAs

## Abstract

Diabetic mellitus (DM) is a significant public health concern worldwide with an increased incidence of morbidity and mortality, which is particularly due to the diabetic vascular complications. Several pivotal underlying mechanisms are associated with vascular complications, including hyperglycemia, mitochondrial dysfunction, inflammation, and most importantly, oxidative stress. Oxidative stress triggers defective angiogenesis, activates pro-inflammatory pathways and causes long-lasting epigenetic changes to facilitate the development of vascular complications. Therefore, therapeutic interventions targeting oxidative stress are promising to manage diabetic vascular complications. Sirtuin1 (SIRT1), a class III histone deacetylase belonging to the sirtuin family, plays critical roles in regulating metabolism and ageing-related pathological conditions, such as vascular diseases. Growing evidence has indicated that SIRT1 acts as a sensing regulator in response to oxidative stress and attenuates vascular dysfunction via cooperating with adenosine-monophosphate-activated protein kinase (AMPK) to activate antioxidant signals through various downstream effectors, including peroxisome proliferator-activated receptor-gamma co-activator 1 (PGC-1α), forkhead transcription factors (FOXOs), and peroxisome proliferative-activated receptor α (PPARα). In addition, SIRT1 interacts with hydrogen sulfide (H2S), regulates NADPH oxidase, endothelial NO synthase, and mechanistic target of rapamycin (mTOR) to suppress oxidative stress. Furthermore, mRNA expression of sirt1 is affected by microRNAs in DM. In the current review, we summarize recent advances illustrating the importance of SIRT1 in antagonizing oxidative stress. We also discuss whether modulation of SIRT1 can serve as a therapeutic strategy to treat diabetic vascular complications.

## Introduction

Diabetes mellitus (DM) is a complicated age-related diseases worldwide (1) and the main characteristics of DM are hyperglycemia and insulin resistance caused by β-cell dysfunction, resulting in increased risk for vascular disease (2–4). Notably, DM patients always accompany with various diseases associated with diabetic vascular complications, such as acquired blindness, atherosclerosis, end-stage renal failure, and neuropathies. Generally, diabetic vascular complications are classified into microvascular and macrovascular complications, the latter of which occur more frequently in patients with diabetes. The central pathological symptoms in macrovascular disease include hypertension, atherosclerosis, diabetic heart diseases, peripheral arterial disease, and stroke. Actually, diabetic patients have two times higher risk of developing stroke and hypertension than those who are healthy adults (5). On the contrary, microvascular complications involve diabetic nephropathy, neurophathy, and retinopathy (6–9). Considering the increased prevalence and the emergent requirement of therapeutic interventions to prevent these complications, it is important to explore the underlying connections between vascular disease and diabetes (10).

Advances in understanding of diabetes have made it clear that abnormal angiogenesis and endothelial dysfunction are two well-documented pathological characteristics of diabetic vascular complications (11–13). In particular, endothelial dysfunction plays central roles in the development of vascular complications, which is characterized by diminished bioavailability of nitric oxide, increased endothelium-derived contracting factors, and the impaired vasodilation. In addition, endothelial dysfunction is accompanied by the accumulation of cytokines and chemokines within the vascular microenvironment (14, 15). Therefore, elucidating the etiology and mechanisms of endothelial dysfunction will facilitate the development of diagnosis, prevention, and treatment even during suboptimal metabolic control of DM.

## Oxidative Stress in Diabetic Vascular Complications

Oxidative stress is manifested as an overload of free radical accumulations, such as reactive oxygen species (ROS), and decreased antioxidants in the microvasculature, which always result in endothelial dysfunction. Importantly, amount of works have demonstrated that oxidative stress is one of the greatest contributors to the pathogenesis of vascular disorders and DM (16–18). For one hand, oxidative stress alters the endothelial signaling transduction and regulates the activation of transcriptional factors in response to redox, and thereby enhances vascular endothelial permeability and leukocyte adhesion. For another hand, oxidative stress contributes to insulin resistance, β-cell dysfunction, and hyperglycemia-mediated cellular injury, leading to the development of DM (16–18).

Specifically, metabolic abnormalities of DM, especially hyperglycemia and hyperlipidemia, can activate NADPH oxidases (NOXs) and endothelial Nitric Oxide Synthase (eNOS), increase advanced glycation end-products (AGE), and interrupt the polyol pathway as well as the mitochondrial respiratory chain, to impinge on oxidative stress (19). Consequently, oxidative stress may impair β-cell function *via* reducing insulin synthesis, hindering proinsulin inclusion, and inducing the apoptotic cell death of pancreatic cells (20). Mitochondrial collapse induced by oxidative stress is another important contributor to insulin resistance. Many high glucose levels associated biochemical pathways, such as glucose autoxidation, prostanoid synthesis, and protein glycation, crumble mitochondrial and promote mitochondria-mediated overproduction of ROS (predominantly superoxide anion) (21). As a result, excess ROS triggers several cellular mechanisms, including polyol pathway, mitogen-activated protein kinase (MAPK), adenosine-monophosphate-activated protein kinase (AMPK), NF-κB signaling pathways, and transcription factors such as forkhead transcription factors (FOXO), Nrf2 and AP-1, to initiate inflammation and deregulate insulin signaling pathways (22, 23). Taken together, therapeutic interventions aiming to reduce oxidative stress, such as anti-oxidative agents, are promising strategies reversing insulin resistance, ameliorating endothelial function and preventing cardiovascular morbidity in patients with diabetes.

## Structure and Regulation of Sirt1

The mammalian sirtuins are nicotinamide adenine dinucleotide (NAD^+^)-dependent histone deacetylases, which regulate numerous cellular processes involved in DNA repair, cell survival and senescence, stress-stimulated metabolism alternations (24, 25). They require NAD^+^ as a cofactor for deacetylation of histone or non-histone substrates and are generally not inhibited by compounds that inhibit zinc-dependent deacetylases. Importantly, increasing reports have demonstrated that the sirtuin family participates in the development of vascular physiology and pathology (26, 27), suggesting the sirtuins as therapeutic targets in DM.

**Figure 1:**
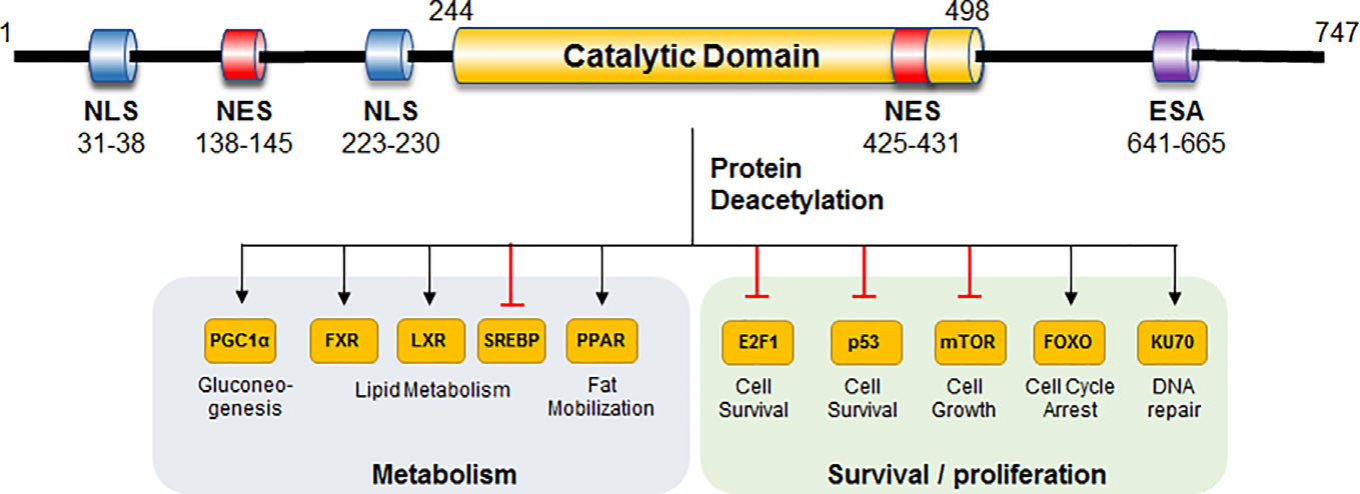
Structure and function of human SIRT1. Important domains and substrates of human SIRT1 are shown, and their corresponding functions are presented. NLS, nuclear localization signal; NES, nuclear export signal; ESA, essential for Sirt1 activity; FXR, farnesoid X receptor; LXR, liver X receptor; SREBP1, sterol-regulatory element binding protein 1.

In mammals, the sirtuin family is comprised of seven members (SIRT1-SIRT7). Among these mammalian Sirts, SIRT1 has been investigated most extensively (28). SIRT1 has 747 amino acids and three independent domains, the central deacetylase domain conservative in species, the N-terminal region containing nuclear localization/export signals, and the C-terminal region containing the essential for SIRT1 activity (ESA) domain (**Figure 1**). Structurally, a substrate and a NAD+ binding pocket exists in the catalytic domain, whereas the regulatory element and binding domain of SIRT1 co-activator/co-repressor distribute in the N- and C-terminus. SIRT1 removes the acetyl groups from multiple cytoplasmic substrates rather than histones, such as peroxisome proliferator-activated receptor-gamma co-activator 1 (PGC-1α), farnesoid X receptor (FXR), liver X receptor (LXR), sterol-regulatory element binding protein 1 (SREBP1), peroxisome proliferative-activated receptor (PPAR) to exert various functions in cellular metabolism (gluconeogenesis, insulin sensitivity, fat mobilization, and lipid metabolism) (29, 30). In addition, SIRT1 also regulate mechanistic target of rapamycin (mTOR), p53, KU70, E2F, and FOXO to affect cell survival and senescence (**Figure 1**).

Multiple studies on the regulation of SIRT1 activity have shown that post-translational modifications (PTMs), such as phosphorylation, S-nitrosylation, and SUMOylation, modulate SIRT1 protein level and its deacetylase activity (31). For instance, SIRT1 contains several sites for phosphorylation (e.g., N-terminus with seven residues and C-terminus with eight residues). In most cases, phosphorylation of SIRT1 mediated by different kinases, such as C-Jun N-terminal kinase 1, casein kinase 2, and cyclin-dependent kinase 1, enhances SIRT1 deacetylase activity and regulates its function in a substrate-dependent manner (31). In addition, SUMO is an ubiquitin like-modifier that exerts opposite effect of ubiquitin and covalently addition of SUMO protein to lysine residues (the so-called SUMOylation) can stabilize proteins. SUMOylation of SIRT1 at Lys 734 also increase its deacetylase activity and protein stability. Conversely, deSUMOylation of SIRT1 by specific deSUMOylating enzyme, sentrin-speciﬁc protease 1 (SENP1), reduces its deacetylase activity (32). Unlike SUMOylation, the covalently incorporating a nitric oxide moiety into Cys 387 and 390 of SIRT1 (S-nitrosylation) significantly reduces the deacetylase activity of SIRT1 toward to a widely reported substrate PGC-1α (33). Furthermore, SIRT1 activity is also regulated by protein-protein interaction. For instance, and deleted in breast cancer 1 (DBC1) and active regulator of SIRT1 (AROS) are negative and positive regulator of SIRT1, respectively. AROS binds to the N-terminus of SIRT1 to increase the SIRT1 activity (34), whereas DBC1 binds to the “essential for SIRT1 activity” (ESA) domain or the SIRT1 deacetylase core domain to inactivate SIRT1 (35). Finally, there are several small chemicals able to modify the activity of SIRT1, such as polyphenol resveratrol, sirtinol, and splitomicin (36). Resveratrol is a stilbenoid and is well-known for its ability to activating SIRT1 to exert its anti-aging, anti-diabetic, and anti-cardiovascular functions. Further works are inspired to discover more SIRT1 PTMs and protein regulators and to explore the possible crosstalk among different SIRT1 PTMs.

## SIRT1 Antagonizes Oxidative Stress in Diabetic Mellitus

**Figure 2:**
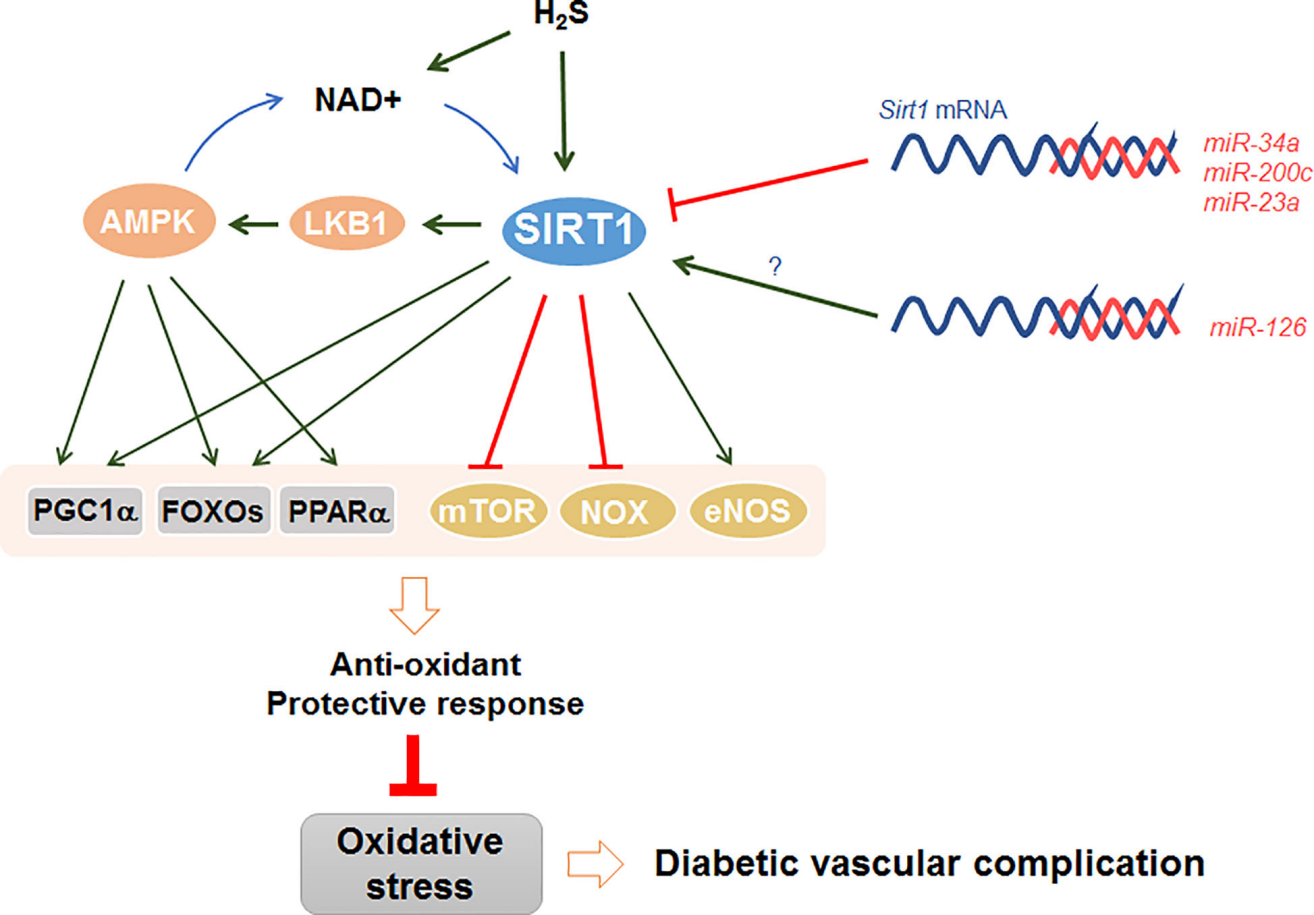
SIRT1 antagonizes oxidative stress in diabetic vascular complications. SIRT1 directly or cooperatively with AMPK to activate various downstream effectors, including PGC1α, FOXOs, and PPARα. SIRT1 also stimulate eNOS and inhibit NOX and mTOR to trigger anti-oxidant protective response. In addition, H_2_S can either activate SIRT1 directly *via* sulfydration, or indirectly *via* increasing NAD+ level. Furthermore, several miRNAs, such as miR-34a, miR-200c, and miR-23a, directly inhibit the mRNA expression of *sirt1* in DM, while other miRNAs including miR-126 promote *sirt1* expression indirectly with unclear mechanism.

Recently published studies have clearly revealed that SIRT1 antagonizes oxidative stress in the pathogenesis of diabetic vasculopathy (37–39). For instance, the downregulation of SIRT1 by hyperglycemia caused vascular dysfunction in DM (40). On the contrary, upregulation of SIRT1 attenuated oxidative stress-induced endothelial senescence in diabetic mice (41). Notably, SIRT1 attenuates oxidative stress to regulate diabetic vascular complications through several important signal mediators, such as AMPK, NADPH oxidase, endothelial NO synthase, mTOR, and miRNAs (**Figure 2**). It seems that SIRT1 relies on the availability of different substrates to regulate cellular oxidative stress.

### SIRT1 Cooperates With AMPK to Antagonize Oxidative Stress in Diabetic Mellitus

AMPK is the main cellular mediator of metabolic stress and is activated by glucose deprivation (42). Importantly, AMPK and SIRT1 both regulate each other and have similar effects on various cellular processes such as cell metabolism, DNA repair, mitochondrial function, and cell growth (43). For instance, AMPK manipulates energy expenditure by increasing the intercellular NAD+ concentration to enhance SIRT1 activity (44). Alternatively, SIRT1 deacetylates and activates LKB1, the upstream kinase of AMPK, to activate AMPK and to suppress ROS production (45, 46). Furthermore, SIRT1 and AMPK share many common targets, such as forkhead transcription factors (FOXOs), peroxisome proliferative-activated receptor α (PPARα), and PGC-1α (43). The joint activation of AMPK and SIRT1 can individually or combinatory activates these downstream effectors to exert antioxidant protective response and to ameliorate oxidative stress in DM (47, 48).

#### Inhibition of Oxidative Stress by SIRT1-AMPK-PGC-1α

PGC-1α is recognized as a key metabolic sensor and regulator in response to malnutrition and hypoxia (49, 50). Upon activation, PGC-1α regulates mitochondrial biogenesis and thus ROS production by controlling many transcription factors to initiate the expression of mitochondrial genes, resulting in reduced oxidative stress (51). PGC-1α is activated either by AMPK-mediated phosphorylation or SIRT1-mediated deacetylation in an NAD^+^-dependent manner (43, 52). Notably, amount of evidence has proved that SIRT1 cooperates with AMPK to enhance the ability of PGC-1α to attenuate endothelial dysfunction *via* stimulating mitochondrial biogenesis (53) and activating gluconeogenic fatty acid oxidation genes (54, 55). In addition, SIRT1-AMPK-PGC-1α pathway exerts its anti-oxidative activity in other diabetic vascular complications including brain complications (56), diabetic cardiomyopathy, and diabetic nephropathy (57, 58). Furthermore, PGC-1α can be directly activated by SIRT1 without AMPK to ease metabolic disorders in high glucose-induced endothelial oxidative damage (59) and Drp-mediated mitochondrial fission in diabetic hearts (60). Therefore, whether SIRT1 regulates the PGC-1α-mediated mitochondrial respiration and ROS production, with or without the help of AMPK activation, rests on a context-dependent way.

#### Inhibition of Oxidative Stress by SIRT1-AMPK-FOXOs

The significance of FOXOs in regulating the metabolic activity of vascular endothelium has been gradually addressed (61). There are four FOXO members in mammals, FOXO1, FOXO3, FOXO4, and FOXO6. SIRT1 deacetylates FOXO1, FOXO3 and FOXO4, and stimulates FOXO-dependent expression of antioxidants to scavenge ROS, such as catalase, manganese superoxide dismutase (MnSOD), and thioredoxin, either positively or negatively (62, 63). For instance, SIRT1 deacetylated FOXO1 to combat oxidative stress in diabetic vascular complications, diabetic nephropathy, and particularly in diabetic cardiomyopathy under the treatment of resveratrol (64–66). Besides, SIRT1 directly deacetylated FOXO3a and promoted the expression of MnSOD and catalase that are involved in stress resistance, which in turn attenuated oxidative stress in diabetic cardiomyopathy and nephropathy (67, 68). Moreover, SIRT1 bound to FOXO4 and increased its transactivation capacity to inhibit NF-κB signaling and inflammation (63). Interestingly, AMPK has also been shown to phosphorylate and activate FOXOs, yet the precise mechanism of AMPK in SIRT1-FOXO pathway remains unclear (44, 64). It seems that AMPK can regulate the function of FOXOs either by direct phosphorylation or indirectly *via* the SIRT1-induced activation.

#### Inhibition of Oxidative Stress by SIRT1-AMPK-PPARα

As a transcriptional factor, PPARα is mainly expressed in many oxidative tissues that require high capacity of fatty acid oxidation and is essential for the regulation of glucose metabolism (69). Additionally, PPARα is able to attenuate oxidative stress and inflammation through repressing NF-κB-mediated gene transcription, as well as increasing NO expression and release (70–72). It has been proved that expression of PPARα was reduced by hyperglycemia, and SIRT1 activated PPARα pathway to reduce ROS in diabetic vascular diseases (73, 74). In addition, AMPK-SIRT1-PPARα was activated by resveratrol to alleviate oxidative stress and endothelial dysfunction in type 2 diabetic nephropathy (58). However, unlike FOXOs, PPARα is not a directly target deacetylated by SIRT1, but its activity can be enhanced by SIRT1 indirectly through the coactivators, such as AMPK and PGC-1α (75, 76). Moreover, various PPAR agonists have been proved to prevent diabetes in the non-obese diabetic mouse model, suggesting the therapeutic function of SIRT1-PPARα axis in DM.

### SIRT1 Regulates eNOS and NOX to Suppress Oxidative Stress

Nicotinamide adenine dinucleotide phosphate (NADPH) oxidase (NOX) family protein are primary sources of regulated production of ROS and play critical functions in the pathogenesis of the macro and micro-vascular complications of diabetes (77). Several important mediators of DM, such as hyperglycemia, oxidized or glycated low-density lipoproteins (LDL) and AGEs, can activate NOX to generate ROS, resulting in cell and tissue injury characteristic of diabetic complications (19). Importantly, the closely connection between NOX and SIRT1 has been gradually recognized, which shows that SIRT1 inhibits NOX to diminish ROS. For instance, resveratrol-mediated activation of SIRT1 inhibited the expression of NOX4 and the NADPH oxidative activity to reduce vascular superoxide production and to ameliorate endothelial dysfunction (78). Besides, dulaglutide, a glucagon-like peptide-1 receptor agonist used to treat type 2 DM, activated SIRT1 to suppress high glucose-induced activation of NLRP3 inflammasome and to downregulate NOX4 expression and the generation of ROS in human umbilical vein endothelial cells (79). In addition, SIRT1 was activated by quercetin to antagonize oxidized LDL-induced endothelial oxidative damage through activating AMPK and inhibiting NOX2 and NOX4 activity (80). Although the detailed mechanisms about how SIRT1 inhibits NOX directly or indirectly though AMPK have not been eliminated, SIRT1 has an antergic action on NOX-mediated ROS production.

Endothelial NO synthase (eNOS) is the primary enzyme producing NO, which participate in mitochondrial biogenesis and has various anti-atherosclerotic functions. Patients with DM demonstrate uncoupled eNOS in blood vessels, resulting in excessive superoxide anion (O2^−^) production and diminished NO availability (81). Up to now, there are extensive connections between SIRT1 and eNOS in counteracting oxidative stress. For instance, in response to calorie restriction, SIRT1 promoted eNOS activity and NO bioavailability *via* the deacetylation of eNOS at lysine 496 and 506 (82, 83). In addition to deacetylation, SIRT1 also positively regulated the phosphorylation of eNOS to affect its ability in reducing oxidative stress and premature senescence (84). Except for SIRT1, AMPK has also been demonstrated to promote NO secretion by increasing eNOS phosphorylation as serine 633, which was another effective way for SIRT1 to overcome caloric restriction and facilitate vascular reconditioning after ischemia *via* activating the AMPK/eNOS axis (85, 86). In addition, SIRT1 enhances eNOS expression as evidenced by that resveratrol induced SIRT1-dependent upregulation of eNOS in endothelial cells, and endothelial-specific overexpression of *Sirt1* led to elevation of eNOS expression (87–89). Furthermore, under resveratrol-stimulated conditions, knockdown of FOXO1 or FOXO3a by siRNAs led to a downregulation of eNOS transcription, implying that SIRT1 activates FOXOs to elevate eNOS (90). Taken together, SIRT1 either enhances eNOS expression or eNOS enzymatic activity to protect against oxidative stress and endothelial dysfunction.

### H_2_S and SIRT1 Interaction Suppress Oxidative Stress

Hydrogen sulfide (H_2_S) is a gasotransmitter playing an important role in physiological conditions, dysregulation of which always occurs in vascular-related diseases. Amount of evidence has clearly demonstrated that interaction between H_2_S and SIRT1 is critical modulator of oxidative stress (91, 92). For one hand, H_2_S limits ROS production *via* directly enhancing SIRT1 activity or expression (93–96). H_2_S promotes the S-sulfydration of two CXXC motifs in the catalytic domain of SIRT1, resulting in elevated protein stability and deacetylase activity (96). H_2_S is also able to activate SIRT1 indirectly by increasing intracellular NAD^+^ levels (97). Furthermore, H_2_S exerts its anti-inflammatory and anti-oxidant effects against diabetic complications *via* stimulating AMPK (92). One possible mechanism is that H_2_S mediates the S-sulfydration of calcium/calmodulin-dependent protein kinase kinase β (CaMKKβ), one important upstream regulator of AMPK, to phosphorylate AMPK and to activate its downstream effector, the SIRT1/PGC-1α axis (98, 99). Taken together, H_2_S and SIRT1 interaction represents a protective mechanism against oxidative stress in DM.

### SIRT1 Regulates mTOR Pathway to Repress Oxidative Stress

The mechanistic target of rapamycin (mTOR) is a nutrient sensor and initiator of cell growth, forming structurally and functionally distinct mTOR complex 1 (mTORC1) and complex 2 (mTORC2) (100). Importantly, mTOR is an important regulator of oxidative stress through regulating mitochondrial biogenesis and enhancing oxidative metabolism *via* PGC-1α pathway (101). Besides, insulin-mediated PI3K/Akt/mTOR signaling axis is one of the most well-known mechanisms in regulating cell cycle, glucose metabolism and endothelial dysfunction in DM (102, 103). Interestingly, under calorie restriction, mTOR was inhibited whereas SIRT1 activity was upregulated (104, 105), suggesting the feedback action between mTOR and SIRT1. Indeed, numerous studies have revealed their possible reciprocal connections. For instance, hepatic SIRT1 deficiency disrupted mTORC2/Akt signaling to cause hyperglycemia and oxidative stress in mice model (106). One possible mechanism is that SIRT1 directly regulated mTOR *via* erythropoietin to protect vascular cells in DM (107, 108). Another example is that SIRT1 activated the mTORC2 signaling *via* activating a cascade of Akt and FOXO phosphorylation, which in turn ameliorated myocardial ischemia or reperfusion injury in DM (103). Together, all the observations imply that SIRT1-mTOR pathway could be potentially therapeutic targets for diabetic vascular complications.

### Regulation of *Sirt1* by MicroRNAs in Diabetic Mellitus

MicroRNAs (miRNAs) are cluster of non-coding RNAs that trigger mRNA degradation and repress translation function mainly by targeting messenger RNAs (mRNAs) (109). Recently, the importance of miRNAs-mediated *sirt1* regulation in diabetic vascular complications has gradually emerged (110). One extensively investigated miRNA was miR-34a (111, 112). Hyperglycemia-mediated oxidative stress recruited p66SHc, which then upregulated miR-34a to impair angiogenesis in endothelial cells *via* directly suppressing the stability of *sirt1* mRNA, leading to the reduced protein level of SIRT1 (112–114). Besides, regulation of miR-34a, SIRT1, and eNOS synthesis promoted kallistatin to inhibit antioxidant gene expression in endothelial progenitor cells (115). In addition to miR-34a, miR-200c and miR-23a were also related to oxidative stress-induced diabetic vascular complications. Hyperglycemia triggered ROS production and upregulated miR-200c expression, which subsequently inhibited the mRNA expression of *sirt1* to facilitate endothelial dysfunction in DM (116, 117). However, unlike miR-34a and miR-200c, increased level of miR-126 could facilitate the expressions of *sirt1* and *sod-2* to enhance oxidative stress and aggravate diabetic vascular complications, with unclear mechanism (118). Aside from miRNAs discussed above, many other miRNAs, such as miR-195, miR-9, and miR-132, have also been reported to target the mRNA of *sirt1* (119–121). Whether they play a role in DM remains to be determined.

## SIRT1 as a Therapeutic Target for Diabetic Mellitus

As discussed above, SIRT1 antagonises oxidative stress *via* different substrates in DM, implying activating SIRT1 as potent therapeutic strategy. Importantly, SIRT1 can be activated directly or indirectly by some naturally polyphenols *in vitro* and *in vivo*, such as resveratrol, quercetin, and catechins (122, 123). For instance, resveratrol is one of the well-studied SIRT1 activators that binds to SIRT1 to promote its substrate binding activity. Resveratrol exhibits various biological functions *via* activating SIRT1 and numerous clinical trials have been performed to test its protective effect against several diseases, such as neurodegeneration, type 2 diabetes, and insulin resistance (123). In addition, some completed phase 1/2 clinical trials have clearly showed that resveratrol is able to improve metabolic and vascular health of diabetes subjects (123). Notably, those natural SIRT1-activating compounds are mostly used as nutraceuticals in management of diabetic vascular complications. Whether their therapeutic effects rely on SIRT1 activation in DM remains to be examined. Considering that most natural compounds possess pleiotropic effects, more selective and specific SIRT1 activators are required, which may facilitate to establish a direct link between SIRT1 activation and human diseases. There are several other SIRT1 activators, such as SRT1720 (an analogue of resveratrol), that have been shown to enhance SIRT1 activity in mammals (36, 124). Whether these activators can exhibit higher specificity and activity than naturally polyphenols, as well as their potentially clinical application, remain to be determined.

## Conclusion

SIRT1 plays a key role in many signaling pathways and regulates various important biological phenomena, especially in metabolism, inflammation, aging, and stress resistance through deacetylating transcription factors and histones. The involvement of SIRT1 in diabetic vascular complications has increased, and the diverse mechanisms of SIRT1 against oxidative stress gradually emerges, suggesting that modulation of SIRT1 activity may be an available therapeutic intervention against diabetes-induced vascular diseases (125). However, many detailed molecular interactions remain unsolved. For instance, a comprehensive understanding of how SIRT1 and AMPK are intertwined in mediating vascular diseases is still needed. It is difﬁcult to parse how speciﬁc interactions between SIRT1 and AMPK contribute to cross-regulation in each vascular complication. Besides, except for SIRT1, there are still other members of the sirtuin family (e.g., SIRT3, SIRT6, and SIRT7) that are active in modulating vascular function (126, 127), which inspires further works to investigate their associations with oxidative stress and diabetic vascular complications. Finally, substantial efforts aiming to develop novel specifically pharmacological SIRT1 activators under active clinical investigation is needed.

## Author Contributions

TM and WQ synthesized and generally organized the manuscript, **Figures 1** and **2**. BL supervised and edited the manuscript. All authors contributed to the article and approved the submitted version.

## Funding

This study was supported by grants from the National Natural Science Foundation of China (91849208, 81972602, 81702909, and 81871114), the National Key R&D Program of China (2017YFA0503900), the Science and Technology Program of Guangdong Province (2017B030301016 and 2019B030301009), and the Shenzhen Municipal Commission of Science and Technology Innovation (ZDSYS20190902093401689, KQJSCX20180328093403969, and JCYJ20180507182044945).

## Conflict of Interest

The authors declare that the research was conducted in the absence of any commercial or financial relationships that could be construed as a potential conflict of interest.
